# The Hypervariable Loops of Free TCRs Sample Multiple Distinct Metastable Conformations in Solution

**DOI:** 10.3389/fmolb.2018.00095

**Published:** 2018-11-13

**Authors:** James E. Crooks, Christopher T. Boughter, L. Ridgway Scott, Erin J. Adams

**Affiliations:** ^1^Graduate Program in Biophysical Sciences, University of Chicago, Chicago, IL, United States; ^2^Department of Computer Science, University of Chicago, Chicago, IL, United States; ^3^Committee on Immunology University of Chicago, Chicago, IL, United States

**Keywords:** molecular dynamics (MD), markov state models, T cell receptor dynamics, adaptive immunity, innate immunity, independent component (IC) analysis

## Abstract

CD4^+^ and CD8^+^ αβ T cell antigen recognition is determined by the interaction between the TCR Complementarity Determining Region (CDR) loops and the peptide-presenting MHC complex. These T cells are known for their ability to recognize multiple pMHC complexes, and for a necessary promiscuity that is required for their selection and function in the periphery. Crystallographic studies have previously elucidated the role of structural interactions in TCR engagement, but our understanding of the dynamic process that occurs during TCR binding is limited. To better understand the dynamic states that exist for TCR CDR loops in solution, and how this relates to their states when in complex with pMHC, we simulated the 2C T cell receptor in solution using all-atom molecular dynamics in explicit water and constructed a Markov State Model for each of the CDR3α and CDR3β loops. These models reveal multiple metastable states for the CDR3 loops in solution. Simulation data and metastable states reproduce known CDR3β crystal conformations, and reveal several novel conformations suggesting that CDR3β bound states are the result of search processes from nearby pre-existing equilibrium conformational states. Similar simulations of the invariant, Type I Natural Killer T cell receptor NKT15, which engages the monomorphic, MHC-like CD1d ligand, demonstrate that iNKT TCRs also have distinct states, but comparatively restricted degrees of motion.

## Introduction

T cells are key components of the adaptive immune system that recognize processed antigens presented on cell surfaces by major histocompatibility complex (MHC) and MHC-like proteins via T cell receptors (TCRs). CD4^+^ and CD8^+^ αβ TCRs recognize antigenic peptides bound to MHC proteins (pMHC), provoking an adaptive immune response to infection, cancer and dysregulated tissue. CD4^+^/CD8^+^ αβ TCRs show both specific and degenerate recognition characteristics. Thymic-selection and homeostatic maintenance in the periphery require that TCRs recognize MHC. However, to provide effective coverage of possible antigens TCRs must be cross-reactive, which has been experimentally demonstrated (Mason, 1998; Wilson et al., 2004; Sewell, 2012). Additionally, to avoid autoimmunity TCRs must distinguish self-peptides from non-self antigenic peptides in the context of MHC presentation, requiring that the TCRs strike a balance between sufficient self-recognition to scan MHCs while avoiding autoimmunity. The key to this balance is the amino acid sequence of the complementarity determining region (CDR) loops of the TCR. These CDR loops are the structural elements that recognize the peptide-MHC surface.

Over two decades of crystallographic work have generated a large database of TCR structures, both free and bound to various foreign and self-reactive peptide-MHC complexes demonstrating significant variation in bound structure that show CDR loop flexibility as vital to TCR cross-reactivity (Armstrong et al., 2008). Reviews of the structural data over the years have concluded, with increasing conviction, that the CDR loops are flexible but in a structured manner distinctly different from the intrinsically disordered regions seen in other proteins (Garcia and Adams, 2005; Rudolph et al., 2006; Armstrong et al., 2008; Baker et al., 2012). Furthermore, general flexibility is restricted to the CDR3 loops, even under extreme changes to CDR3 loop length (Reiser et al., 2002). The issue of flexibility becomes a matter of when this flexibility exists, expressed in the tension between the induced fit and pre-existing equilibrium hypotheses. The induced fit model argues for initial weak binding between the TCR and MHC, which allows for a conformational change to make stronger contacts resulting in a higher affinity interaction with recognized peptides (Boniface et al., 1999; Wu et al., 2002). Biophysical measurements including mutational studies, ITC, and kinetic measurements have supported this view (Wu et al., 2002; Gakamsky et al., 2007). Of more direct interest for the present article, structural studies have suggested a strong role for the CDR1β and CDR2β germline-encoded loops, which have shown the least rearrangement upon binding, in MHC recognition (Feng et al., 2007; Ishizuka et al., 2008). This would provide the necessary initial bias toward MHCs required for the induced-fit model.

An alternative “conformer” model suggests that cross-reactivity could instead be driven by the existence of multiple CDR loop conformational states of the free TCR, which could recognize different peptide-MHC ligands so that specificity is controlled by a combination of specific contacts and the relative equilibrium populations of different conformational states (Holler and Kranz, 2004). These two models are difficult to distinguish biophysically as loop dynamics are difficult to capture even with techniques capable of resolving time-dependent dynamics as in Scott et al., though the measurements did substantiate the use of computational methods (Scott et al., 2012). Computational analysis of the free A6 TCR provides strong support for the existence of distinct states in solution, where clustering the CDR3α and CDR3β loops using RMSD as a dissimilarity metric showed multiple distinct conformations of the loops (Scott et al., 2011, 2012). Notably, CDR3α showed slow motions between two clusters of conformations, one cluster resembling a bound state of A6, and the other cluster distinct from the bound state, while CDR3β appeared to sample multiple dissimilar conformational clusters. These clusters, along with recent NMR experiments showing that the CDR3β loop of 2C is still mobile in the 2C-QL9-L^d^ complex, have motivated a “conformational melding” hypothesis combining aspects of induced-fit and conformer models (Hawse et al., 2014). CDR3 loop flexibility has also been computationally observed in the TCR-pMHC bound state over a vast array of peptides (Knapp et al., 2014). Determining which of these models best describes TCR dynamics is critical to our understanding of TCR-pMHC recognition, particularly the mechanisms behind cross-reactivity and specificity. Understanding how individual amino acids can affect these TCR-specific properties may help with a more rational design of personalizeable immunotherapies.

A key requirement of the conformer models and conformational melding hypotheses is the existence of distinct crystal-like states in the unbound TCR's dynamics. In order to probe these dynamics at time- and length-scales inaccessible by experimental methods, we utilized molecular dynamics (MD) simulations and analyzed the subsequent trajectories using a Markov State Model (MSM) framework. Molecular dynamics is becoming ever more prominent in molecular biology, and the formalism and usage of MSMs has moved quickly from a humble beginning focused on alanine dipeptide to a promising potential in drug discovery (Pan and Roux, 2008; Bowman et al., 2015; Meng et al., 2016; Gu et al., 2017).

**Figure 1:**
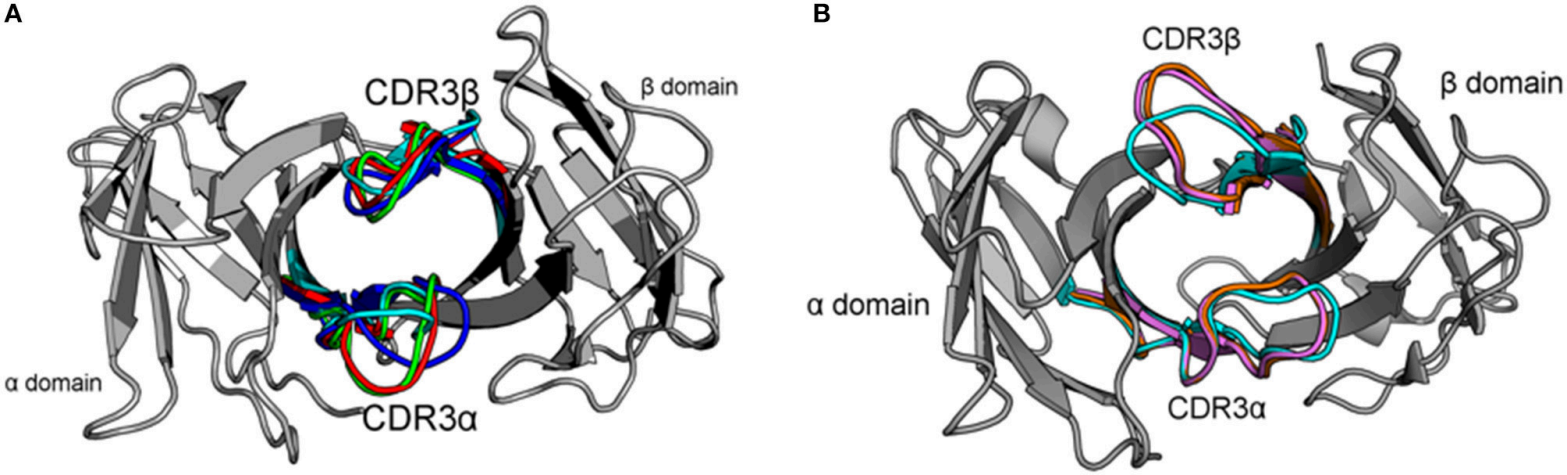
Crystal structures of the 2C and NKT15 TCRs highlight their hypothesized dynamics and the starting points used for simulations. **(A)** Variable domains (gray) of 2C shown from the perspective of the pMHC surface. CDR3 loops shown for the unbound structure (cyan, PDB: 1TCR), bound to H-2K^b^/SIYR (red, PDB: 1G6R), H-2K^b^/QL9 (green, PDB:2CKB), and H-2L^d^/QL9 (blue, PDB:2OI9). **(B)** Similar view of NKT15 variable domains (gray) with CDR loops shown for the unbound structure (cyan, PDB:2EYS), and bound to CD1d presenting αGalCer (orange, PDB: 3HUJ) or C20:2 (pink, PDB: 3VWJ).

We have run extensive simulations of the free 2C TCR, as well as simulations of the free Natural Killer T cell receptor NKT15 to directly address flexibility. 2C is a well-studied TCR known to display significant cross-reactivity and with extensive crystallographic data available (Figure 1A) (Garcia et al., 1996, 1998; Degano et al., 2000; Colf et al., 2007). We have generated a total of 3μs of data across 10 trajectories of 2C, providing a significantly larger data set than has been previously available to study the solution state dynamics of a TCR. Further, we have used the Markov State Model formalism to cluster the loop dynamics in a kinetic fashion, providing crystal-like states that distinguish stable conformations from transitions and directly identifying transitions between conformational states. In accordance with previous work on A6, we observe significant flexibility in both CDR3α and CDR3β, with CDR3α showing a broad energetic well representative of a non-binding state and CDR3β showing multiple meta-stable states with local equilibria.

In contrast to CD4^+^ and CD8^+^ αβ T cells, type I Natural Killer T cells (NKT) recognize lipids presented by the monomorphic MHC-like molecule CD1d. Type I NKT TCRs are considered to be “semi-invariant,” as they are generated through VDJ recombination as per standard αβ TCRs but use a heavily restricted Vα and Vβ chain repertoire. This restriction, along with orders of magnitude faster binding kinetics, higher affinities, and rigid binding conformations in crystal structures have led them to being considered “innate-like” (Figure 1B) (Rossjohn et al., 2012). Importantly, mutational studies implicate the CDR2β and CDR3α loops in driving the NKT interaction with CD1d and the canonical antigen, αGalCer (Wun et al., 2012). The conserved binding footprint, innate-like kinetics, and stronger reliance on germline encoded interactions implies that type I NKT TCRs should demonstrate reduced flexibility and simpler dynamic behavior, particularly in the CDR3 αβ loop, compared to classical CD4^+^/CD8^+^ TCRs. The restricted binding footprint in the NKT-Ag-CD1d system suggests that the NKT TCRs should serve as “innate-like” counterpoints to CD4^+^/CD8^+^ αβ TCRs, despite using the same fundamental protein architecture. To test this model, we have simulated 1μs of free NKT15 dynamics across 10 trajectories. Surprisingly, we observe flexibility and meta-stable states in both the CDR3α and CDR3β loops of NKT15. However, in contrast to 2C, the dynamic behavior of NKT15's loops is simpler in that for each loop, the major motions can be well-captured by a single degree of freedom.

## Materials and methods

### MD simulations

All simulations were carried out using the Amber14 package. Input coordinates were prepared from PDB files 1TCR (2C) and 2EYS (NKT15), truncated to the variable domains and prepared using pdb4amber preprocessing scripts. These structures were solvated with TIP3P waters in an octahedral unit cell at 12 angstroms, neutralized with NaCl at 150mM concentrations, and parameterized using the AMBER99SB force field and Joung/Cheatham ion parameters using xleap (Ponder and Case, 2003). Two rounds of 2000 steps of minimization were carried out, first with restraints on the protein, and then secondly without restraints. These minimized states were the initial seeds for each of the 10 trajectories run out for each of 2C and NKT15. Each trajectory was set to 300 K through initial velocity randomization, and allowed to equilibrate in NPT for 10 ns using a Langevin thermostat and the Amber Monte Carlo barostat at 1 atm, allowing the trajectories to diverge independently (Hoover, 1985). All data presented in the analysis was collected following the 10 ns equilibration stage, with each trajectory run for an additional 300 ns (2C) or 100 ns (NKT15) using SHAKE to allow 2 fs time steps (Ryckaert et al., 1977). Calculations were performed using the CUDA-enhanced pmemd Amber module (Götz et al., 2012; Salomon-Ferrer et al., 2013).

### Data analysis

Raw simulation data was processed using cpptraj to re-image the system and extract protein data (Roe and Cheatham, 2013). Structure alignments and RMSD calculations were carried out using VMD (Humphrey et al., 1996). Hydrogen bonds were determined using a 3.2 Å distance cutoff and 20° angle cutoff in VMD. Solvent accessible surface area (SASA) was calculated in VMD with a probe radius of 1.4 Å, with only the CDR3 loops, residues 92–97 and 205–210, included in the calculation. Further data processing used custom Python scripts with trajectory featurization and data handling provided by the MDTraj library (McGibbon et al., 2015). We used the MSMBuilder3 library to perform time-lagged independent component analysis (tICA), clustering, and Markov State Model generation as described in their sections (Beauchamp et al., 2011). Kernel density estimates were calculated using the gaussian_kde_ module from the Scipy library; kernel bandwidth was selected using Scott's Rule (Scott, 2015).

In our analysis, we perform all tICA decompositions using a fixed choice of time-lag at 5 ns to make the analysis more comparable across decompositions. We then calculate distances in this tICA space while clustering using the standard Euclidean metric after projecting the data frames onto the first 16 tICA degrees of freedom. We chose 16 degrees of freedom as they account for >90% of the energy of the eigenvalues of the tICA decomposition. All clustering was done using the K-medoids algorithm. We clustered the CDR3α data into 16 clusters, and CDR3β data into 32 clusters. We chose 16 and 32 clusters because the cluster medoids gave reasonable coverage of the state space shown by the first two degrees of freedom, permitted more clusters for coverage of any significant unobserved parts of state space present in higher degrees of freedom, and were low enough numbers to achieve reasonable convergence of the microstate Markov model in the case of CDR3β. The CDR3α model did not converge, as described in the results.

After clustering, a “microstate” Markov model is constructed by estimation of a state transition matrix through maximum likelihood estimation (MLE). MLE finds the transition matrix that has the greatest likelihood of generating the observed state transitions. The estimation works from the raw data by counting transitions between clustered states over a chosen time-lag. That is, if we are estimating a Markov model with time-lag τ and the data is in state 1 at time *t* and in state 2 at time *t*+τ, then we count that as a transition from state 1 to state 2. To determine the best choice of time-lag, we constructed Markov models for multiple time-lags, making a final choice of model using time-lags where the slowest few degrees of freedom converge to stable values (Figure S1).

**Figure 2:**
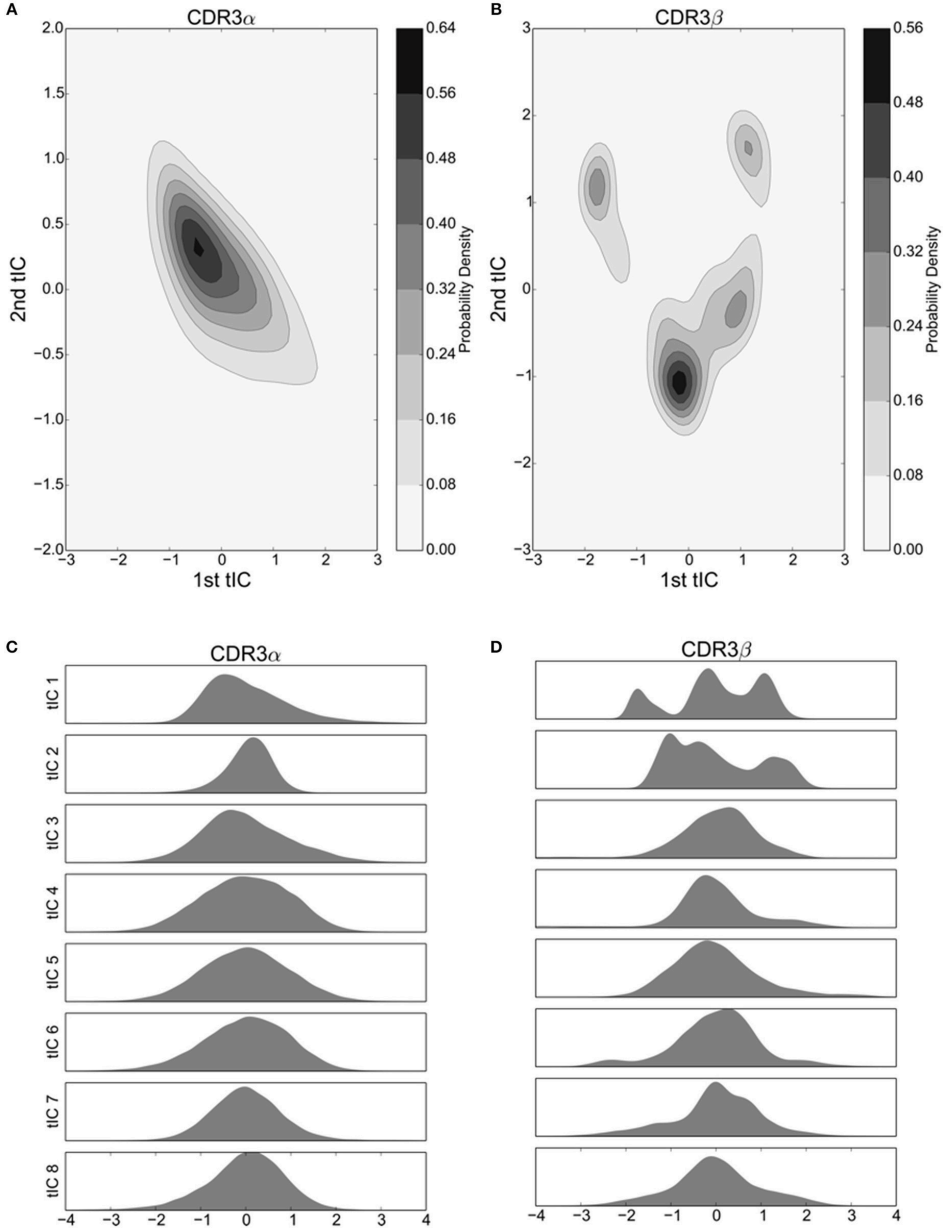
The first 8 tICs of the tICA-decomposed dihedral angles show relatively simple motions of the CDR3α loop, and more complex behavior for the CDR3β loop for the 2C TCR. **(A,B)** 2-D Kernel Density Estimate of the simulation data projected onto the first two degrees of freedom discovered by tICA for the CDR3α and CDR3β loops, respectively, using a 2-D Gaussian kernel. The KDEs estimate the probability density function for finding a randomly selected frame in a region of conformational space described by the tICA degrees of freedom. **(C,D)** 1-D probability density graphs of the first eight tICA degrees of freedom for CDR3α and CDR3β, respectively, using a Gaussian kernel.

The final “macrostate” Markov model, which our analysis focuses on, is determined from the microstate model by Perron Cluster Clustering Analysis (PCCA), which clusters together the microstates in the microstate model. The microstates are joined to form macrostates, where the clustering is determined by maximizing the stability of the new macrostate, i.e., maximizing the probability of self-transitions. Macrostate assignment was performed using the PCCA+ algorithm, which improves on the results of PCCA. The CDR3β microstate model was built with a time-lag of 8 ns, and clustered into four macrostates as evidenced by both the relaxation timescales (Figure S1) and the four identifiable local maxima of the first two tICA degrees of freedom (Figure 2B). Further detail regarding tICA and Markov State Modeling can be found in the accompanying Supplemental Methods section.

## Results

### tICA analysis shows stable conformations and low dimensional motion

To understand the dynamical motions of the 2C TCR, we ran 10 molecular dynamics simulations for 300 ns (totaling 3μs of simulation time) of the 2C α and β variable domains using the coordinates of the 1TCR PDB as initial coordinates. We studied the conformational changes of the CDR3α and CDR3β loops individually by analyzing their backbone dihedral angles under the tICA decomposition. The tICA decomposition is similar to Principal Component Analysis (PCA), in that it looks for linear combinations of degrees of freedom (here, the dihedral angles) that better describe the data while holding each combination in the set to be independent of the others. PCA finds new combinations of variables that capture a maximal amount of variance in the data. Unlike PCA, the tICA algorithm attempts to find combinations of variables that describe slow variations in the data over a given timescale chosen by the data analyst. A formal description can be found in the Supplemental Methods section, but intuitively, the tICA algorithm takes a dataset and a chosen timescale as inputs and returns a set of combinations of the dihedral angles that are independent of one another and display long-lived behaviors. The tICA decomposition has been previously shown to effectively find slow degrees of freedom for domain motions by itself (Naritomi and Fuchigami, 2011), and the slow degrees of freedom found by tICA act as an effective pre-processor for MD data in protein folding studies (Schwantes and Pande, 2013).

We applied the tICA decomposition to the phi and psi angles of CDR3α and CDR3β independently, each with a time-lag of 5 ns. Considering only the first two degrees of freedom resulting from this analysis, we see a local maximum of the probability distribution for the CDR3α loop (Figure 2A), while there are four regions with local maxima for the CDR3β loop (Figure 2B). These islands of locally high probability are long-lived regions of conformational space that are frequently visited by the simulation, suggesting that these conformations are relatively stable, indicating the existence of stable conformational states.

An outstanding question drawn from crystallographic studies asks how free are the motions of the CDR3 loops (Garcia and Adams, 2005; Armstrong et al., 2008)? Are they weakly structured with a large number of degrees of freedom to move in, or are they tightly choreographed, moving in distinct conformational states? To address these questions, and confirm the value of our two-dimensional distributions, we consider the probability distributions of the first eight tICA degrees of freedom. Eigenvalues of the tICA decomposition show that the first eight degrees of freedom capture the vast majority of information in the decomposition, and the first 16 degrees of freedom capture substantially all of the information (data not shown). CDR3α shows an asymmetric distribution in the first and third tICA degrees of freedom, and a highly peaked distribution in the second tICA degree of freedom centered away from zero (Figure 2C). The remaining tICA degrees of freedom are more Gaussian with means near zero, suggesting that CDR3α has some mild internal structure to its motions, with at most only the first three tICA degrees of freedom capturing interesting behavior. CDR3β shows significantly more interesting behavior in its first two tICA degrees of freedom, both of which show multiple peaks, while the remaining degrees of freedom show much more Gaussian-like appearances (Figure 2D). This strongly suggests CDR3β's motions are primarily captured by the first few, particularly the first two, tICA degrees of freedom, indicating highly structured motions.

### Markov state model of CDR3β shows discrete metastable states

Next, we clustered the frames via k-mediods into a microstate model and estimated microstate models for CDR3α and CDR3β. The CDR3α microstate models do not converge over the timescales analyzed (Figure S1A), and the trajectories are of insufficient length to extend to larger timescales. This implied that CDR3α has a very slow degree of freedom that is not sufficiently explored in the simulation. We address this later in the section on reverse simulations.

**Figure 3:**
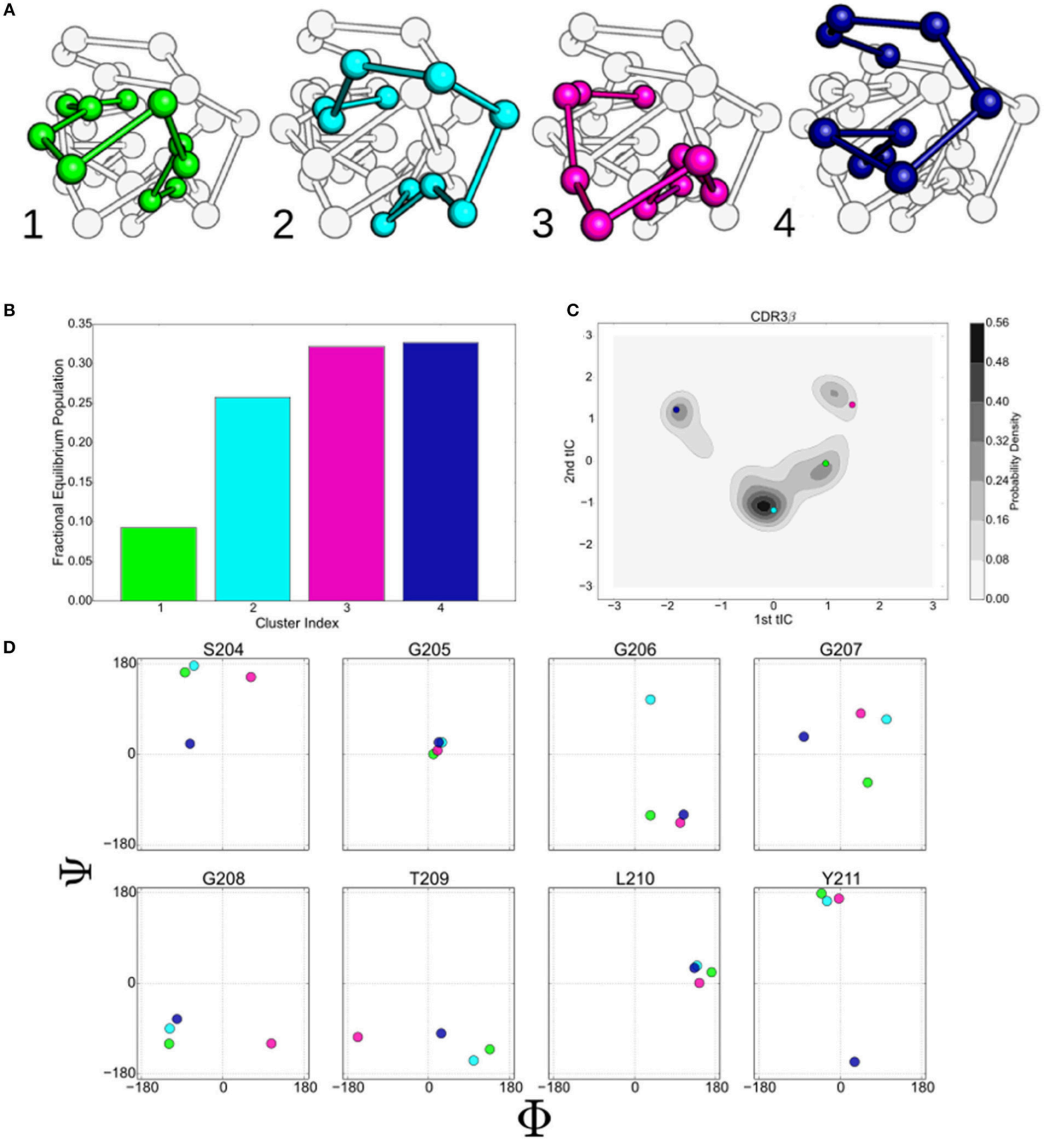
The states identified by the k-mediods clustering of the tICA decomposition provide insight in to the complex motions that the 2C CDR3β loop displays. **(A)** Ball and stick model of the CDR3β loop showing the centroids of the four macrostate clusters determined by the MSM. Centroids were determined by finding the orientation that minimizes the distance to all other members of the cluster under the tICA projection distance. **(B)** Equilibrium populations of the four clusters, determined by eigenvalue analysis of the macrostate MSM. **(C)** Projection of the centroids onto the first two tICA dimensions overlaid on the kernel density estimates of the projected data. **(D)** ϕ/ψ backbone angles of eight residues along the CDR3β loop. Colors are consistent throughout for state 1 (green), 2 (light blue), 3 (purple), and 4 (dark blue).

The three slowest implied timescales of the CDR3β models separate out from the faster timescales when the implied timescales converge (Figure S1B). This supports a four-state model of the CDR3β system and agrees with the four high-probability islands observed in the 2D projection of the data using tICA, leading us to build a four-state model for the macrostate MSM. Centroids, the orientations that are in the centers of the clusters, are shown for each state in Figure 3A. All four states are well-populated at equilibrium (Figure 3B), with the fourth state showing the largest equilibrium population. State 4 is particularly notable, as it appears to be involved in a hydrogen bonding interaction with the CDR3α chain, described further in a later section. All four states show distinct structural differences and are clustered near the centers of the wells in tICA space (Figure 3C), with large shifts in most dihedral angles of the loop observable between the different centroids.

The backbone ϕ/ψ angles of the eight central residues of the CDR3β loop are shown in Figure 3D. G205 and L210 show minimal variation between the centroids, suggesting that flexibility at these positions is not required to generate the observed collection of metastable states. Diversity is seen in both of the angles of G207, while S204 separates out the state 1 and 2 centroids from the others along the ψ and ϕ angles respectively. G206, G208, and Y211 primarily separate a single centroid orientation from the other three along a single ϕ or ψ angle, while showing minimal variation in the non-separating angle. T209 appears to separate centroids along the ϕ angle, however variation is seen under re-clustering of the original microstate clusters, while the behavior of the other angles is stable, implying that T209 is flexible but does not meaningfully describe the different states (data not shown).

**Figure 4:**
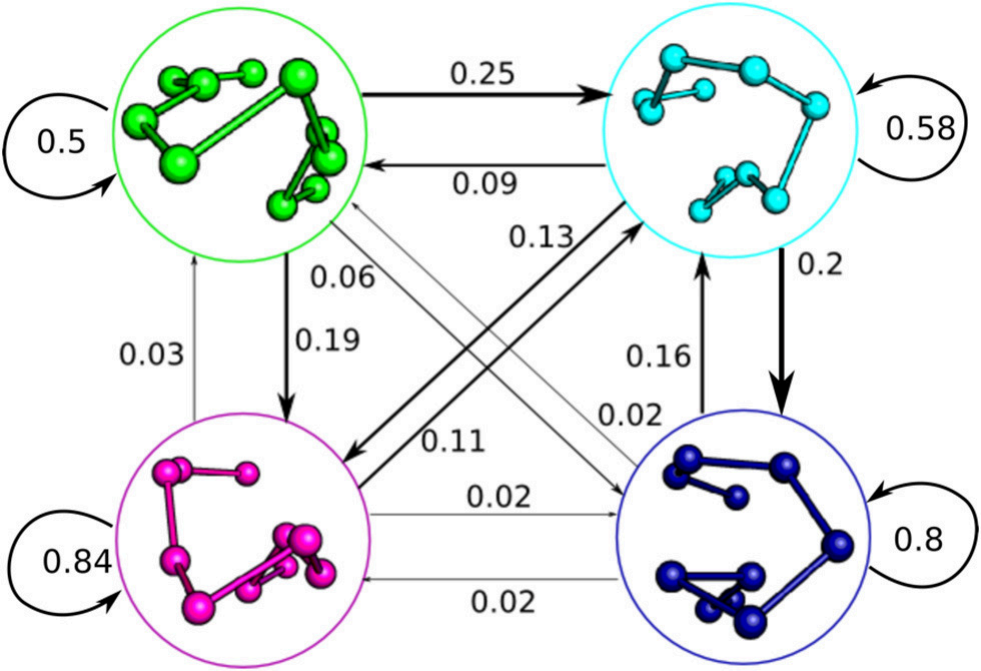
Macrostate Markov State Model of the 2C CDR3β, showing the complex dynamic behavior the loop adopts in solution. The Markov model was built with a time-lag of 115 ns, distinct from the 5 ns time-lag used for the tICA decompositions. State clusters are represented by their centroids as initially described in Figure 3A, and jump probabilities are described by arrows labeled by the probability of that state transition occurring in a 115 ns time step. Arrow size is proportional to jump probability.

The macrostate Markov model of CDR3β shows distinct pathways between the different metastable clusters and differing levels of metastability in the states, with states 3 and 4 showing strong metastable behavior, while states 1 and 2 are only weakly stable (Figure 4). Despite the relative instability of state 2, it acts in a hub-like fashion, with the largest rates into states 1, 3, and 4 all coming from state 2. Rates into state 2 are also highly relative to all other state transitions, with the exception of the state 1 to state 3 transition, which shows similar magnitude to the state 1 to state 2 transition. The other transitions show much lower flux rates, so that state 2, although only weakly metastable, acts as a central metastable intermediate. This high flux into state 2 accounts for the high population observed in the equilibrium distribution of the state despite the weak stability. State 1 is also weakly metastable but does not have a counter-balancing inward flux, leaving it as a simpler weak metastable state, which accounts for its low equilibrium population. State 1 has a large outward flux to both state 2 and state 3, with the most significant in-flow coming from the hub-like state 2, positioning state 1 as an alternate pathway to access the much more stable state 3. State 4 only shows significant exchange with the hub-like state 2 and shows strong stability and high equilibrium population similar to state 3.

### CDR3α and CDR3β loops of type I NKT TCRs have metastable states

Unlike CD4^+^ and CD8^+^ αβ TCRs, type I NKT αβ TCRs recognize lipids presented by CD1d, a monomorphic MHC-like protein (Rossjohn et al., 2012). NKT TCRs do not show significant variation in their bound state footprint, and crystal structures show comparatively little movement between free and bound conformations, despite variation in the chemical structures of the presented lipids (Kjer-Nielsen et al., 2006; Pellicci et al., 2009; Wun et al., 2012). Type I NKT TCRs show significantly higher binding affinities than CD4^+^/CD8^+^ TCRs, and have binding kinetics that suggest an innate-like response (Rossjohn et al., 2012). As they use the same immunoglobulin architecture as standard CD4^+^/CD8^+^ TCRs, we investigated the unbound dynamics of the NKT TCR as a comparison to the dynamics of the 2C system. We ran 10 independent trajectories of the NKT15 TCR for 100 ns, collectively totaling 1μs of data.

**Figure 5:**
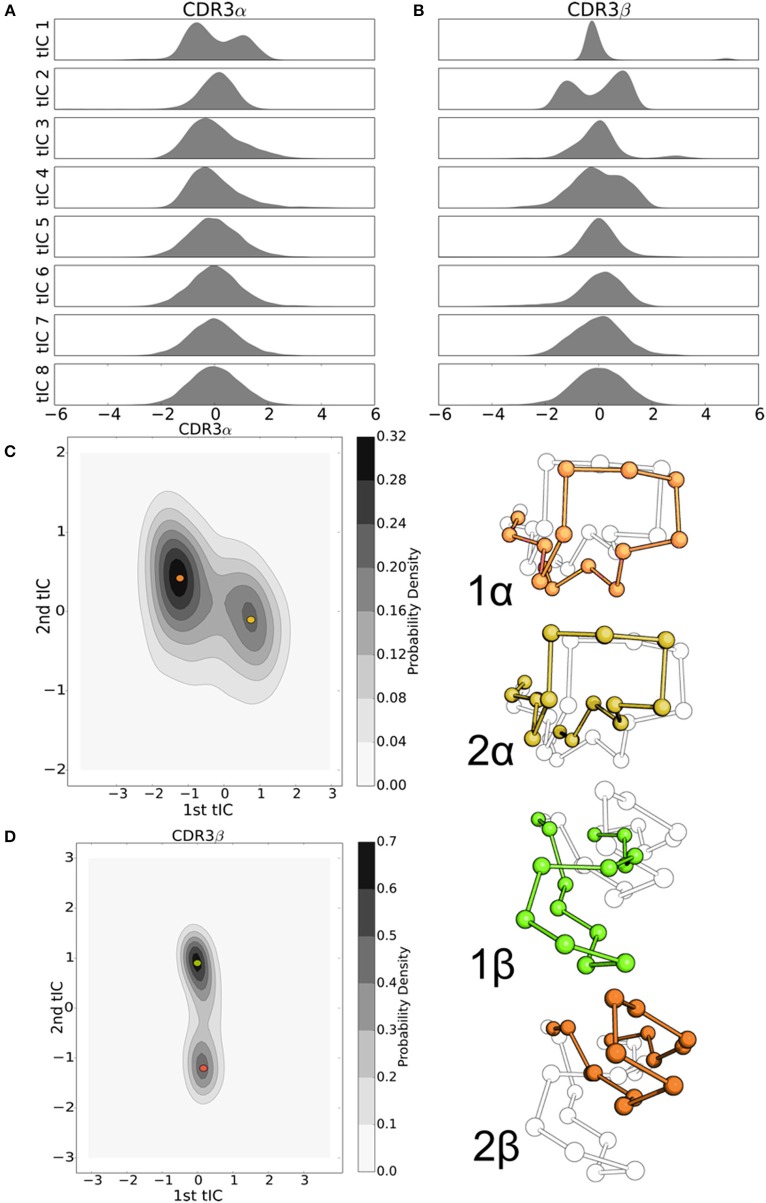
tICA decomposition and k-mediods clustering of the resulting data highlight the significant, but simple, motion of the NKT15 TCR CDR loops. **(A)** Probability distributions of the NKT15 CDR3α and CDR3β conformations projected onto each of the first eight tICA degrees of freedom, computed by a 1-D kernel density estimate with a Gaussian kernel. **(B)** 2-D probability distribution of NKT15 CDR3α projected on the first two tICA degrees of freedom; selected conformations from the simulation are shown in orange and gold and overlaid on the probability distribution plot. **(C)** As in panel **(D)** for the CDR3β loop with selected conformations shown in light green and ochre. Probability distributions were computed by a 2-D kernel density estimate with a Gaussian kernel over all collected trajectory data.

We applied the tICA decomposition to the backbone dihedral angles of the CDR3α and CDR3β loops of NKT15 with a time-lag of 5ns. Similar to the 2C TCR, the tICA decomposition is indicative of low-dimensional, structured motions. Most of the tICA degrees of freedom consist of Gaussian motions around a mean of zero, thus consisting of thermal motion, with only one degree of freedom for each loop showing multiple peaks that suggest metastable conformational regions (Figures 5A,B). Plotting the density estimates of the first two degrees of freedom for each loop, we find that both loops show two distinct high probability regions separated by lower probability transition regions (Figures 5C,D). In both CDR3α and CDR3β, the two local probability maxima are separated along a single axis, so only a single degree of freedom is responsible for the transition between these high-probability regions. Furthermore, in both systems, one of the high-probability regions shows a much higher probability relative to the other, suggesting the existence of a single major local energy minima, and a kinetically nearby metastable state with higher energy. In contrast to 2C, we observe distinct metastable regions in both systems, although CDR3β is much simpler in NKT15 than in 2C, with only two metastable states separated along a single degree of freedom, implying that NKT15's motions are more restrained than 2C.

### CDR3α and CDR3β loops interact in 2C through hydrogen bonds

**Figure 6:**
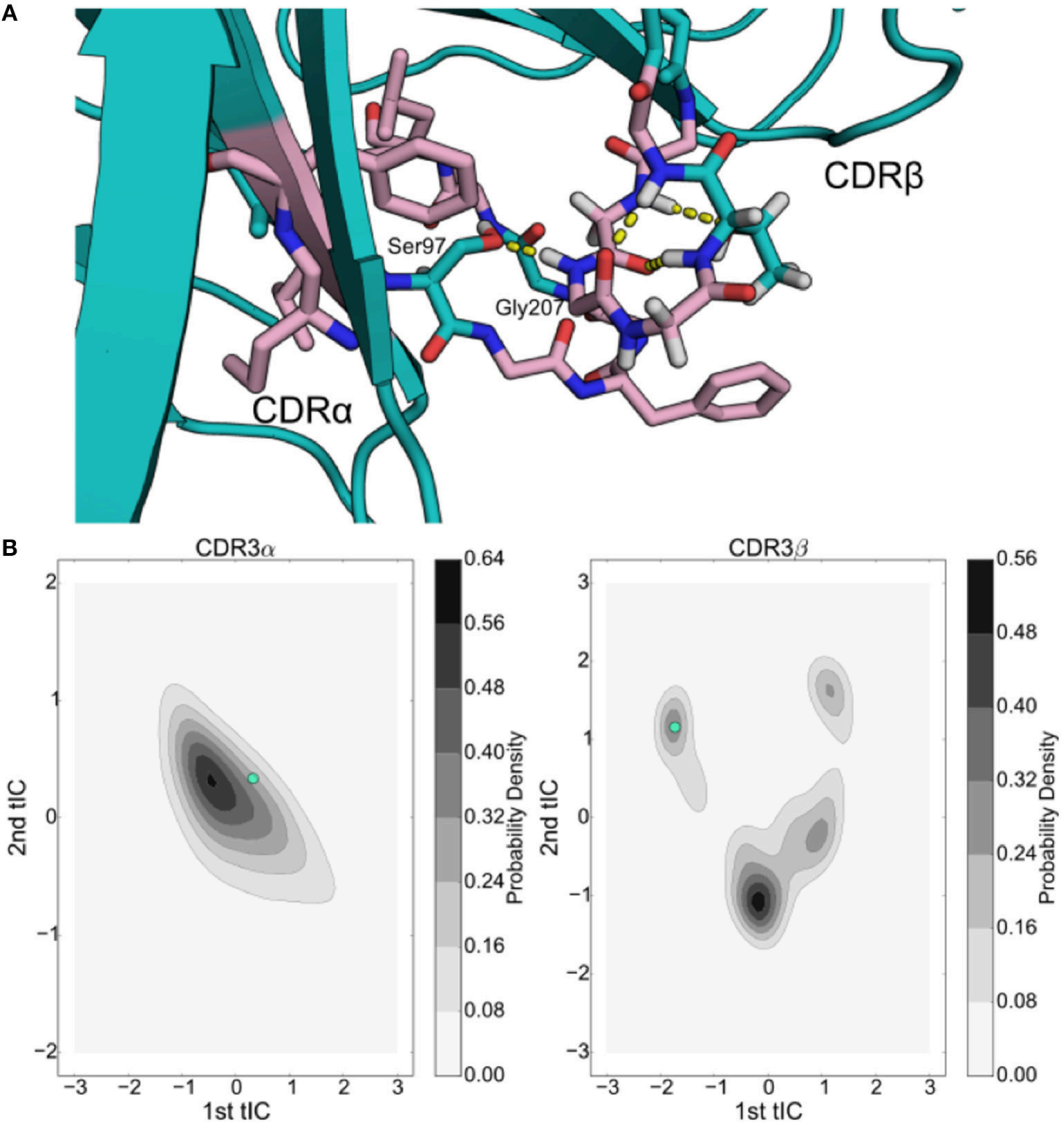
Inter-loop hydrogen bonding plays a significant role in the simulations of the 2C TCR, comprising one of the 4 stable wells of the CDR3β tICA decomposition. **(A)** Structural rendering of the 2C hydrogen bond interaction between Ser93 and Gly207 with the Vα domain shown on the left and the Vβ domain shown on the right. Surrounding hydrophobic residues are shown in pink. **(B)** Projection of the data frame onto the tICA projections of the CDR3α (left) and CDR3β (right) loops overlaid in cyan.

Previous work has shown that there is weak, if any, coupling between the overall loop dynamics of CDR3α and CDR3β loops in the A6 TCR, yet the DMF5 TCR showed highly correlated motion (Scott et al., 2012; Ayres et al., 2016). For 2C, we observe direct hydrogen bond interactions between the CDR3α and CDR3β loops of 2C when CDR3β adopts meta-stable state 4. In two of the ten trajectories of 2C, the CDR3β loop adopts a conformation that permits a hydrogen bond between the sidechain of Ser93 on CDR3α and backbone of Gly207 on CDR3β (Figure 6A). The CDR3α loop's conformation that permits this bond is near the high-probability region observed in the tICA projection and may account for some of the long-tail spread observed in the first tICA degree of freedom for CDR3α (Figure 6B). The CDR3β loop of 2C appears to only be able to form this bond in state 4 where the CDR3β loop is oriented to make the Gly207 backbone contact with the CDR3α Ser97. The hydrogen bond demonstrates significant stability, appearing in 25% of frames assigned to state 4. The persistence of this interaction and the specificity of the orientation required to allow it accounts for the high equilibrium population of state 4 in the Markov state model. As the model relies on kinetic information to determine equilibrium populations, rather than just directly observed conformations, the model indicates that this hydrogen-bonded state will tend to be a high-population, dominant state over a much longer sampling time.

This conformation is further stabilized by multiple intra-loop polar contacts and a hydrophobic “shell” that protects the hydrogen bonds from solvent interactions. In addition to the inter-loop contact between Ser93 and Gly207, in the sample frame we observe a CDR3α-CDR3α hydrogen bond between Ser93 and the backbone of Gly94, as well as three CDR3β-CDR3β intra-loop hydrogen bonds between Thr209 and Gly206 (Figure 6A). Surrounding these hydrogen bonds are numerous hydrophobic residues that can shield the hydrogen bonds from solvent, which has been shown to enhance stability in other contexts (Fraser et al., 2010, 2011). There are nine hydrophobic residues within 6 angstroms of either Ser93 or Gly207, creating a hydrophobic shell around the inter-loop hydrogen bond and shielding some of the intra-loop interactions as well. This hydrophobic region demonstrates significant stability, as transitions out of this state once entered are not seen in the trajectories where it occurs. This “hydrophobic collapse” conformational state is both structurally and kinetically separated from the other conformations, notably looking unlike known bound states of 2C, and potentially acting as a hydrophobically driven “off” state that reduces the overall affinity of the TCR by stabilizing a binding-incapable state.

At the same time, the hydrophobic side chains that contribute to the stability of state 4 may explain the instability of states 1 and 2 in which the CDR3β loop is more extended and thus more solvent accessible. The increased solvent exposure of the hydrophobic sidechains will create unstable conformations, leading the CDR3β loop to “search” for a conformation that once again buries the hydrophobic residues, leading to the transition-state behavior of states 1 and 2 where the CDR3β loop is frequently sampling, possibly unsuccessful, transitions out of the conformational state. State 3, which is a strongly stable state similar to state 4, does not display the same “hydrophobic collapse” properties. Instead, this state appears to have a more significant solvent accessible surface area (SASA) with states 1 and 2, the more “bound-like” states, having a SASA of 1358.43 and 1430.10 Å^2^, respectively, state 3 having a SASA of 1463.42 Å^2^ and state 4 having a SASA of 1262.89 Å^2^. While state 3 is not “collapsed” it is conformationally distinct from states 1 and 2 and crystallographic structures, suggesting this may again be a driven “off” state, driven by ordered waters that have been previously suggested to be critical in TCR-pMHC interactions (Armstrong et al., 2008; Holland et al., 2012).

### Simulations reproduce CDR3β bound crystal structure orientations

**Figure 7:**
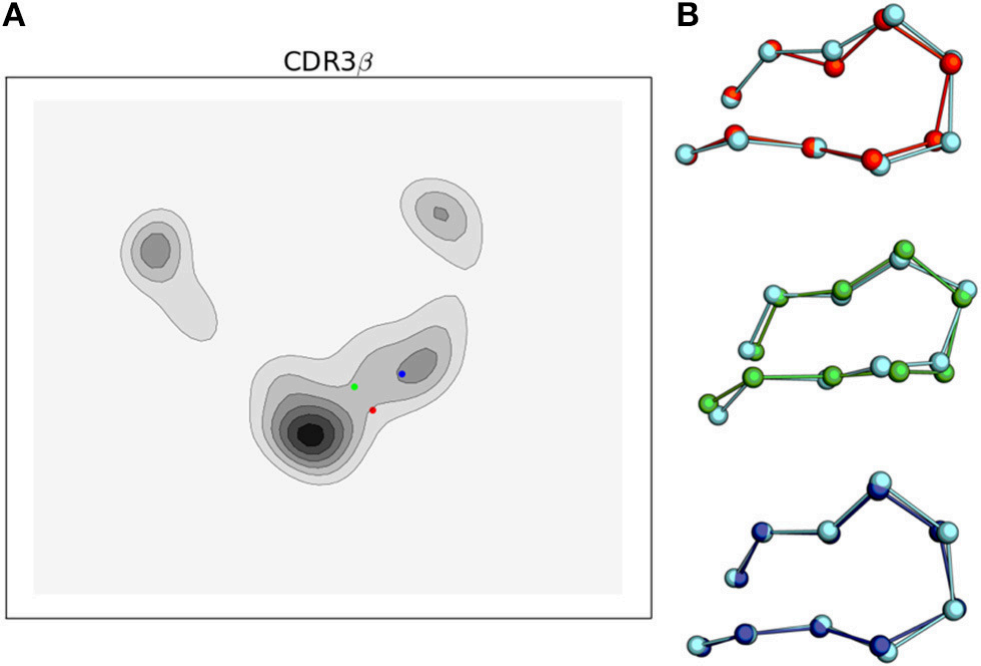
Simulations of the 2C TCR CDR3β loop recapitulate bound crystal structure conformations and support the conformational melding hypothesis based on their location in the tICA projections. **(A)** tICA projections of the bound 2C CDR3β loop conformations for 2C bound to H-2K^b^/SIYR (red), H-2K^b^/dEV8 (green), and H-2L^d^/QL9 (blue) overlaid on the 2-D probability density. **(B)** Ball and stick render of the CDR3β bound crystal structures overlaid with the nearest simulation frame by RMSD of the Cα's after aligning the β variable domain. Simulation data is shown in cyan.

We are able to compare our results with experimentally determined crystal structures in two ways. First, as the tICA projection matrix can be applied to existing data sets, we projected the CDR3β loop conformations of three bound structures of 2C in complex with H-2K^b^/SIYR (PDB 1G6R), H-2K^b^/dEV8 (PDB 2CKB), and H-2L^d^/QL9 (PDB 2OI9) onto the first two tICA degrees of freedom (Figure 7). We omit CDR3α projections because no bound states of the CDR3α loop are found in the simulation trajectories, implying either a much slower transition time as observed for A6 in Scott et al. (2012), or that the conformation of the CDR3α's bound state is unfavorable without the environment of the peptide-MHC.

Projecting the bound conformations onto the first two tICA degrees of freedom, we find that H-2K^b^/SIYR and H-2K^b^/dEV8 both appear near the most frequently observed region of the tICA conformational space but are themselves in low probability regions that appear to be transition regions between two meta-stable states. This indicates that although the bound conformations for these antigens are closely sampled in solution, they are unlikely to directly be the result of selection from a pre-existing equilibrium. However, they are kinetically close to two well-populated meta-stable states, making it plausible that if a binding event is initiated from either of these two meta-stable regions, then CDR3β will be able to rapidly find the correct orientation observed in the bound state. In contrast, the bound conformation for the alloreactive H-2L^d^/QL9 falls into the region corresponding to state 2 of the MSM, which is the lowest equilibrium population state of the model. Intriguingly, both antigens that use the H-2K^b^ MHC fall into the transition-like region, but nearer to the hub-like state 2, while H-2L^d^/QL9 falls into a distinct region and biologically presents in a different context than H-2K^b^.

### Reverse simulations indicate slow CDR3α dynamics

In our main dataset, CDR3α did not transition to a bound-like conformation in any of the 10 trajectories. This strongly suggests that the bound conformation lives in a stable local energy minima with slow kinetics. To test the stability of the bound state, we ran an additional 10 trajectories of 2C, initialized with the coordinates of the bound state for 2C bound to H-2K^b^/SIYR. Trajectories were run for 100 ns each, collecting an aggregate of 1μs. CDR3α remained near the bound conformation for the entirety of all 10 trajectories, in line with the hypothesis that the bound state is a stable local well. Because no transitions are observed in any trajectory, we are unable to construct a Markov State Model of CDR3α, however the data indicate that CDR3α is stable in the bound conformation independent of the environment of the peptide-MHC, and the kinetics of transitions between these states are very slow. This is in line with observations of A6, where simulations yielded only a single transition of CDR3α in an aggregate data set of 460 ns (Scott et al., 2011), suggesting that slow CDR3α dynamics may be a general feature of CD4^+^/CD8^+^ TCRs.

## Discussion

The flexibility and dynamics of the CDR loops of T cell receptors have long been a topic of speculation and interest. Crystallographic work has long demonstrated the existence of multiple loop conformations in the final bound state of the CDR loops and indicated that loop flexibility must necessarily play a role in cross-reactivity. Here, we have used the Markov State Model framework to show that in 2C's CDR3β loop, there exist clusters of conformations that are distinct and exist independent of the environment of the final binding state, and that these conformations are much broader even than those variations observed in the known crystal structures of 2C, our model system. We have shown that these individual states, made of many kinetically related conformations, are inherently stable in a fashion that makes them fitting of the term state, and there exists a distinctive structure in the movements of the loops between these states. Previous computational work demonstrated the existence of distinct clusters of conformations in the unbound A6 TCR, and provided evidence for a slow mode of motion in the CDR3α loop and faster, more diverse, motion in the CDR3β loop (Scott et al., 2012). Our results find good agreement with this work, suggesting a common behavior; that of slower, simpler movement in the CDR3α loop and faster, more complex motion in the CDR3β loop, for CD4^+^/CD8^+^ αβ TCRs. We have furthermore demonstrated the stability of these clusters, showing them to be true local minima, providing distinct conformational groups that can potentially act as a source of initial conformations from which selections can be made, in either a conformational selection or conformational melding model of TCR-pMHC recognition.

The tICA decomposition is a powerful tool for understanding the complexity of the motions we observe. Previous work, both computational and crystallographic, has firmly established the flexibility of the CDR3 loops in CD4^+^/CD8^+^ αβ TCRs, but it has been difficult to understand how well-structured those flexible motions are, that is, are the motions precise and organized through specific degrees of freedom, or are the loops more like a rope, able to flex anywhere along its length? With the tICA decomposition into linear, orthogonal degrees of freedom, we can characterize these motions by the number of orthogonal degrees of freedom that meaningfully contribute to the state transformations, in the case of 2C CDR3β, we observe two orthogonal degrees of freedom captured by the tICA decomposition that reveal evidence of substates and probability densities that are distinctly non-Gaussian. Thus, 2C's CDR3β loop moves, with respect to its internal motions, through a two-dimensional space, and has a restricted flexibility. Even more strikingly, we see that with a tICA decomposition of the available data for NKT15, both CDR3α and CDR3β are described by a single tICA degree of freedom. The Cα at the tip of the CDR3β loop of NKT15 shows a larger variation in its location in real space than the corresponding measurement of 2C's CDR3β loop, however NKT15 is less flexible in that it has fewer degrees of freedom, forcing it to adopt simpler motions than those available to 2C.

This difference in the dimensionality of flexible motion of the TCRs is a qualitative demarcation between 2C and A6 on one hand, and NKT15 on the other. More recent work from Ayres et. al. characterizing the A6 and DMF5 TCRs suggests a spectrum of dynamic behaviors for similar TCR sequences (Ayres et al., 2016). In the present work, only one of 2C's bound states falls into the locally most probable region (QL9-Ld antigen), while the other two bound states appear in a lower probability transition region between two wells. This supports the conformational melding hypothesis; there are clear clusters of conformations that would be capable of more quickly finding the proper bound state, but the actual bound states are not so likely that the binding mechanism is well described as conformational selection. However, Ayres et. al. find that the A6 and DMF5 TCRs better fit the conformational selection and induced-fit models, respectively (Ayres et al., 2016). It is possible that the use of tICA and Markov models could yield similar results for these TCRs, with the originally distinct conformational selection and induced-fit models converging toward this middle ground of conformational melding in the context of higher-dimensional analysis.

We can roughly partition agonist αβ TCR kinetics into two classes, those which have slow off rates, and those with on rates faster than diffusion where analysis of re-binding events have been shown to effectively predict signaling (Govern et al., 2010). In the more classical, slow off rate case, “local search” would explain the slow observed binding kinetics, as put forward in the conformational search and conformational melding hypotheses. Conformational melding effectively argues that the search is local, requiring cooperation with the pMHC, and thus must be seeded by a conformation that is initially selected from a set of equilibrium conformations. These initial conformational seeds may make sufficient contacts with pMHC for a local search and refinement of the CDR loop structure can then commence from these seeds, with final contacts dependent on the bound peptide. In this model, TCRs can search through pMHCs quickly and efficiently and initiate response only if a stable final conformation is found. In the induced fit model, conformational search on the pMHC surface may be too slow for sufficient specificity, i.e., a very slow interaction may lead to more non-specific responses, whereas in the pure conformational selection model, there is less room for promiscuity, as there are only so many compatible states that are kinetically distinct and possible in solution. The observed state clusters in our Markov model provide distinct initial states in accordance with the melding hypothesis for the 2C TCR, where a local search starting from these initial states induced by the pMHC can lead to a specific interaction.

On the other hand, the innate-like kinetics of type I NKTs would suggest simpler motions, which are apparent in the tICA decomposition of the NKT15 simulation data. The crystallography of type I NKTs demonstrates little variation in binding orientation. Unlike 2C, the footprints of type I NKT TCRs are nearly identical across different antigens (Pellicci et al., 2009; Wun et al., 2012), which is fitting with the faster kinetics. The need for faster kinetics and thus simpler loop dynamics can potentially explain the reduced selection of variable domains: the reduced selection has been evolutionarily selected specifically for the tendency to create simpler loop dynamics, while still using recombination to make minor adjustments to the enthalpic contacts and potentially alter the equilibrium distribution of states. As we observe two states for each of the CDR3α and CDR3β loops in NKT15, it is reasonable to postulate that these alternative states may act as simple “off” states that do not permit binding, thus acting to modulate the overall affinity.

A major outcome of 2C's flexibility is the creation of the hydrogen bond interaction between CDR3α and CDR3β and the hydrophobic region that stabilizes the interaction. It is likely this state is binding-incapable, as the cluster conformations differ sharply from the bound conformations present in crystal structures, which suggest a dual-role for CDR3β in both MHC recognition and overall affinity adjustment. The hydrogen bonded state 4, and the less well-characterized, but similarly stable state 3 in our MSM of CDR3β appear to be “off” states, whose equilibrium populations would control affinity by altering the probability that the TCR is binding competent or binding incompetent. A similar role has been suggested for CDR3α in the context of A6 due its slow motions (Scott et al., 2012). If these states are also reachable in the bound system, they may also adjust the off-rates depending on how accessible they are. On the other hand, states 1 and 2 divide the bound conformations by MHC, suggesting that CDR3β conformations contribute to MHC recognition as well as peptide specificity. Despite the length of our simulations, transitions out of the hydrogen-bonded state are not observed, which limits our understanding of the state dynamics and limits the quantitative value of the CDR3β MSM. Nonetheless, the qualitative results, the existence of four distinct conformational clusters, is clear.

While the primary focus of this research is on the kinetics of unbound T cell receptors, much work has been done in the field to characterize the thermodynamics of the TCR-pMHC interaction. This work has mostly centered around the roles of entropy and enthalpy in TCR binding. One might expect, and in some cases it has been shown, that there is a high entropic penalty upon TCR binding and the majority of the positive interactions are enthalpic (Boniface et al., 1999; Willcox et al., 1999). However, there has been significant research demonstrating that favorable entropic interactions, via a relatively stiff TCR or ordered waters on a pMHC surface, can be the driving force behind a TCR-pMHC recognition event (Armstrong et al., 2008; Holland et al., 2012; Madura et al., 2013). Here we find that our results strike a middle ground for the role of entropy and enthalpy in adaptive immune recognition. While the existence of stable unbound CDR3 states that are structurally similar to bound TCR-pMHC conformations suggests a low entropic penalty upon binding, states 3 and 4, the most stable states in our Markov model, would be entirely excluded when 2C is bound to pMHC. These results suggest that 2C's promiscuity for different ligands bound to *H-2K*^*b*^ may be the result of relatively low entropic penalties upon binding, and therefore the dependence on favorable enthalpic interactions is lessened, which leaves room for a wider range in the peptides recognized. However, without a more complete picture of the interaction, i.e., simulations of the peptide-MHC complex, we cannot form a complete picture of the thermodynamics of this interaction.

Finally, we note that the existence of these slow dynamics and long-lived metastable states indicates a need for significantly longer trajectories and larger data sets. We have contributed a large data set for a single TCR, which we believe to be the largest set of trajectories for a free TCR that deals with only a single system, and thus is comparable across trajectories, as well as allowing for independent trajectories to evolve. Much work has largely used 100 ns or shorter trajectories, often with fewer trajectories, with trade-offs between deeper sampling of a particular phenomenon or broadly sampling many comparable systems forced by technological and resource constraints (Borbulevych et al., 2009; Scott et al., 2012; Knapp et al., 2014). Using MSMs to knit together multiple trajectories into a larger picture and taking advantage of GPU-enhanced calculations to greatly extend the size and scope of simulations offers a much more comprehensive picture for single systems. While T cell receptors have been the primary focus of MD simulations in immunology, peptides bound to MHC have been studied to further improve dynamic structural insights (Reboul et al., 2012). These results, coupled with clear evidence that crystal structures are unable to tell the full story, will hopefully provide other researchers with the impetus to further utilize molecular dynamics simulations in their work (Holland et al., 2018).

## Author contributions

EA, LS, and JC designed research. JC performed research. JC, CB, LS, and EA analyzed data. JC, CB, and EA wrote the paper.

### Conflict of interest statement

The authors declare that the research was conducted in the absence of any commercial or financial relationships that could be construed as a potential conflict of interest.
