# Supplementary material for: The Hypervariable Loops of Free TCRs Sample Multiple Distinct Metastable Conformations in Solution
Source: Front Mol Biosci. 2018 Nov 13;5:95. doi: 10.3389/fmolb.2018.00095 (PMC6243104; doi:10.3389/fmolb.2018.00095)
Supplement: Supplementary file 1 [file Data_Sheet_1.pdf]

Supplemental Information

Supplemental Methods

tICA Decomposition

The tICA decomposition is a time-dependent variation on Independent Components Analysis, a technique originally introduced in the signal processing literature to solve the Blind Source Separation problem. Similar to PCA, tICA constructs a new orthogonal basis set for the data, usually for the purpose of dimensionality reduction, as used here, by projecting the data onto the linear subspace spanned by the first few basis elements of the tICA decomposition.

Formally, PCA solves the eigenvalue problem

$$\Sigma = P \Lambda P^{-1}$$

where Σ is the covariance matrix of the data. The eigenvector matrix P is, under suitable normalization, an orthonormal basis set for the PCA and the eigenvalues along the diagonal of Λ represent the amount of variance of the original dataset that is captured by the corresponding eigenvector.

However, capturing variance is not a suitable goal for finding useful reaction coordinates of a protein system. Instead of maximizing variance, tICA finds a new basis set which maximizes the autocorrelation of the time-series data when projected onto the new basis-set. This is a more useful metric for protein dynamics, where we are interested in the slow motions representative of conformational changes rather than the fast motions that are primarily thermal noise.

By finding degrees of freedom with maximal autocorrelations, tICA instead finds degrees of freedom which kinetically separate regions of local stability in the conformational space. The tICA degrees of freedom capture the slow dynamics of the protein system. As described in Schwantes and Pande, 2013, the tICA algorithm formally solves the generalized eigenvalue problem given by

$$C^{(\Delta t)}\mathbf{v} = \lambda \Sigma \mathbf{v}$$

Where C is the correlation matrix of the data with time-lag Δt, Σ is the standard covariance matrix of the data, and λ and $\mathbf{v}$ are eigenvalue/eigenvector pairs. The set of all such eigenvectors form the tICA degrees of freedom.

Markov State Model Construction

Markov state models are a discrete approximation to the Master equation that governs a dynamical system, as described in Prinz et al., 2011. Thorough introductions can be found in Pande et al., 2010. Essentially, we partition the data frames into discrete states, initially via a clustering algorithm that operates directly on the simulation frames.

The final macrostate MSM transition matrix is re-estimated directly from the data using the same MLE procedure as the microstate model, with the data frames assigned to the macrostate cluster determined by the PCCA process. The robust variant of PCCA, termed PCCA+, was used to perform the clustering (Deuflhard and Weber, 2005).

Placing the Metastable States in the Context of MHC Recognition

The focus of this paper is on the dynamics of the TCR in the unbound state, but this naturally leads to the question "Do these states have direct relevance to possible binding modes on the pMHC surface?". In Figure S2 we superimpose the four metastable TCR states on three peptide-MHC complexes (H-2Kb/SIYR, H-2Kb/dEV8, and H-2Ld/QL9) shows that these metastable CDR3 conformations are likely only found in the unbound state. The alpha chains (left) extend too deeply in to the binding pocket, clashing with all three peptides. For the beta chain, state 3 (purple) appears to clash with the alpha-2 helix, while states 2 (green) and 4 (blue) appear to clash with all three peptides. While state 1 (cyan) does not appear to have any steric clashes, this binding mode is actually further away from the pMHC complex while maintaining a similar footprint to the crystallized structure (in white). While these results suggest the centroids of the unbound metastable states show low relevance to the bound state, a higher-level analysis focused on determining how these metastable states might interact with pMHC and different peptides in the binding pocket is a logical future direction of this work. A more rigorous treatment with docking algorithms and full simulations of the TCR-pMHC complex, while not relevant in the scope of this paper, will certainly provide exciting insights to the field.

Supplemental Figures

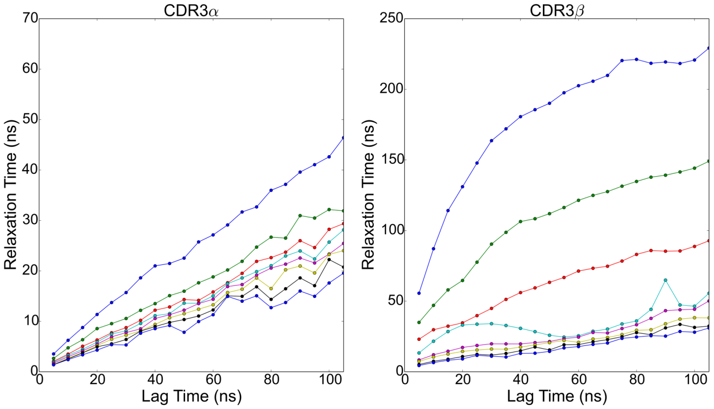

Figure S1: Implied timescales/relaxation timescales derived from eigenvalue analysis of microstate MSMs for CDR3α (A) and CDR3β (B) loops.

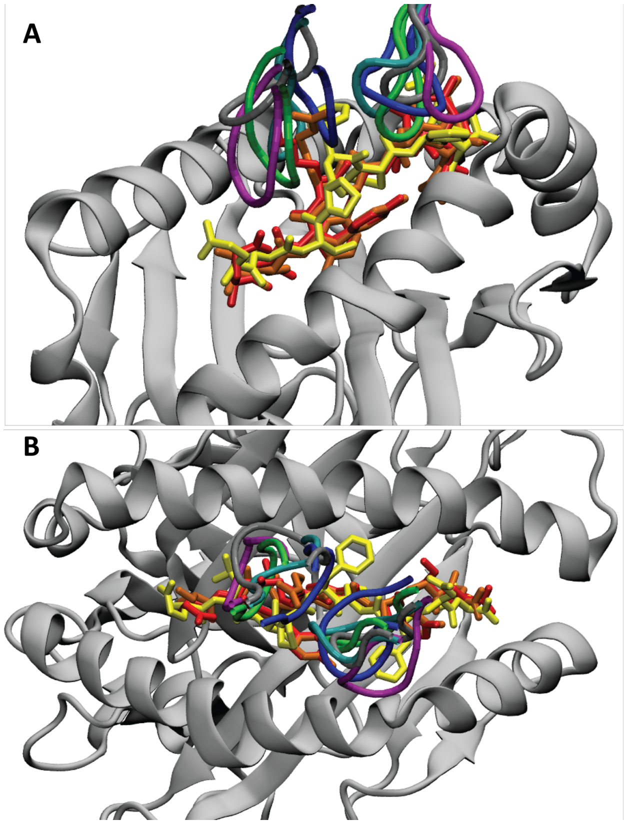

Figure S2: Centroids of the metastable Markov States superimposed on to a TCR-pMHC complex from the side (A) and top (B) show that the alpha loops (left) and beta loops (right) clash with both the peptide and the MHC helices without the assistance of more sophisticated computational techniques like docking or molecular simulation. State 1 is shown in cyan, state 2 in green, state 3 in magenta, state 4 in blue, and the 1G6R crystal structure is shown in white for the MHC and grey for the CDR3 loops. Peptides are from the 1G6R (orange), 2OI9 (yellow), and 2CKB (red) PDBs.
